# Spiritually significant hallucinations: a patient-centred approach to tackle epistemic injustice

**DOI:** 10.1192/bjb.2023.17

**Published:** 2024-04

**Authors:** Rachel J. Cullinan, Angela Woods, Joanna M.P. Barber, Christopher C. H. Cook

**Affiliations:** 1 Cumbria, Northumberland, Tyne and Wear NHS Foundation Trust, Newcastle upon Tyne, UK; 2 Newcastle University, Newcastle upon Tyne, UK; 3 Durham University, Durham, UK; 4 Birmingham and Solihull Mental Health NHS Foundation Trust, Birmingham, UK

**Keywords:** Spirituality, epistemic injustice, religious hallucinations, hallucinations, chaplaincy

## Abstract

This article uses three fictitious case vignettes to raise questions and educate on how clinicians can appropriately approach patients experiencing spiritually significant hallucinations. Religious hallucinations are common but are not pathognomonic of mental illness. They are often intimate experiences for the patient that raise complex questions about psychopathology for clinicians. When assessing a patient with religious hallucinations it is important that clinicians hold at the centre that person's personal experience and create a safe space in which they are listened to and epistemic injustices are avoided. Involvement of chaplaincy services is important not just to support the patient but also to ensure that as clinicians we seek support in understanding the religious nature of these experiences.

## Clinical scenarios

### Scenario 1

A 26-year-old man became a Christian in the past year and has subsequently spent many hours each day reading the bible and praying. During one of these sessions a month ago he began hearing the devil telling him that he is sinful and deserves to die in pain. He has been praying to God and asking for forgiveness but has only ever heard the devil's voice. He asks his doctor why God has abandoned him and what he can do to be forgiven. He then asks his doctor to pray with him.

### Scenario 2

A 50-year-old woman describes herself as a prophet and is experiencing voices which talk about the end of the world. A priest she has spoken to supports this interpretation. She and her husband both had profound conversion experiences at university and travelled across Africa to teach and evangelise. On return to the UK, she found a job as a teacher. She is a respected member of the local church and popular with her students; however, her work colleagues are concerned that she is teaching about her prophecies in lessons. She believes her spiritual experiences are a gift to be cherished.

### Scenario 3

A 20-year-old Muslim man has become increasingly withdrawn and will not leave his room. He describes battling with voices that tell him to kill his father. He knows it is wrong to do this and has been desperately trying to resist them. He believes these voices are jinns and has been prayed for by his imam, which helped initially but now he is worse again. Family members tell him he is not praying hard enough. He is frightened and feels out of control. He pleads to be allowed to see the imam again.

## Introduction

These three case vignettes were created based on clinical experience (but modified to maintain anonymity) to reflect some of the wide variety of spiritually significant hallucinations that may be experienced. They give rise to the following questions, which we will address here:

- How do you approach history taking in cases with religious hallucinations?
- Is it important to discriminate between religious experiences and psychopathology?
- How and when should chaplaincy/spiritual-care services be involved?

From this article, we want readers to recognise the importance of how patients are asked about spiritually significant hallucinations, to be aware of epistemic injustice and to appreciate the benefits for patients and clinicians of involving chaplaincy services.

## Review of the literature and gaps in evidence

Auditory verbal hallucinations (AVHs) are perception-like experiences of a voice without external stimuli and usually beyond a person's control.^1^ Religious AVHs are those in which the identity or content of the voice(s) relate to traditional religious beliefs, persons or stories, such as the jinns in scenario 3. The prevalence of religious hallucinations varies worldwide, but they remain relatively common in most countries. Occurrence appears to increase in areas where religion is an important part of public and private life,^2^ although this finding is complicated by lack of clarity about what is defined as ‘religious’.^3^ A wider perspective includes experiences described as ‘spiritual’; these may have cultural relevance, but also can be entirely idiosyncratic or subjective. A common theme from both religious and spiritual definitions is that of transcendence – the relationship with non-material, spiritual or divine order understood as being above and beyond the natural.^4^ The term ‘spiritually significant’ hallucination was introduced by Cook et al based on how patients identified the voices they hear according to context, content or identity of the voice, etc.^5^

Hallucinations can be core symptoms of a variety of mental illnesses; however, not all voice-hearing is indicative of psychopathology.^6^ Luhrmann describes ‘imaginal relationships’ within which God ‘feels real’. These occur over time following intense spiritual practices such as prayer or meditation and may be experienced by an individual or shared and/or fostered by the whole community. Luhrmann distinguishes these from hallucinations seen in psychosis and calls them ‘sensory overrides’, through which perceptions or phenomena are experienced, such as hearing a voice.^7^ De Leede-Smith & Barkus compared the phenomenological characteristics of voices heard by clinical (with confirmed psychotic disorder) and non-clinical populations.^8^ The most notable psychological factor which distinguished clinical from non-clinical auditory hallucinations was the negative emotional valence for clinical individuals. It is important to remember that the experience of hearing voices can be meaningful and life-enriching whether the voices are part of a mental illness or not. The clinician therefore needs to take a sensitive history and conduct a careful mental state examination to avoid diagnosing a mental illness when one is not present.^5^

We know that spirituality and religion can aid, or impede, coping with illness and adversity.^9^ In general, positive religious coping is associated with better outcomes, and negative religious coping with poorer outcomes, for most forms of mental illness. Positive religious coping includes such things as forgiveness, benevolent reappraisal of circumstances in the light of faith, and drawing on the support of religious communities and practices. Negative religious coping, sometimes also referred to as spiritual struggles, involves negative reappraisals (for example, believing that illness is a punishment from God), belief in demonic causation of problems, and interpersonal conflict within a faith community. For religious people experiencing AVHs or delusions of any sort, finding positive religious coping strategies should be facilitated wherever possible alongside other treatment options. There is evidence that clinicians may have a positive role in mobilising these positive coping resources;^10^ however, this is under-researched and sometimes also held back by a lack of understanding from the faith community.^11^

## Reflections and considerations

### The patient perspective (J.M.P.B.)

First, it can be difficult for a patient to trust anyone. This can lead to isolation, making the content of their experiences even more compelling to them, and may also impede the development of therapeutic relationships with mental health staff. My own experience of struggling to trust anyone began long before I had any contact with mental health services. When I first had problems of this sort, I sought help from the church. Repeated exorcisms helped for a while, but things kept coming back even worse than before. I felt blamed, guilty and cynical of the whole process and left the church. I felt extreme fear, guilt and shame associated with my experiences.^12^ I imagine that the patients in scenarios 1 and 3 might also feel this way. In scenario 2, the patient may well be suspicious that the clinician will disbelieve her, just as everyone else has done. In all three scenarios there is great distress and each patient needs to know that their psychiatrist is on their side, trying to understand. In scenarios 1 and 3, simple reassurance and promise of help will in itself be helpful. Their questions and requests are a sign of their desperation, a cry from the heart. In scenario 2, the patient has been threatened with losing her job and she may well have been subject to ridicule, and acknowledging this will help make a connection with her.

Second, speaking to a priest or chaplain may be very important to a patient. The clinician should therefore recognise the limitations of their role and offer a chaplaincy referral at the earliest opportunity. For scenarios 1 and 3, the authority of a priest of the appropriate faith could provide great comfort and strength. Scenario 2 is, in a way, more difficult since the patient is not actually asking for help, but she too might appreciate chaplaincy support.

Third, if psychiatric illness may be involved, great sensitivity needs to be shown when discussing this with the patient. In all these situations, simply explaining experiences away as illness and of no value can be painful and cause alienation. However, the concept of illness can be helpful, if and when people are ready to accept it. It can ease the burden of thinking that it is all their fault, that they have not prayed hard enough or had enough faith, especially in scenarios 1 and 3. In the longer term, it can aid recognition of when they need to seek help. Even then, religious issues could again become overwhelming and insisting on a medical interpretation might be unhelpful. The content of religious/spiritual experiences may be remembered for a long time after recovery.

Whatever a clinician says, thinks or does, a religious interpretation will probably always be there, in the back of a patient's mind. Thus, religious difficulties need to be addressed alongside any psychiatric treatment. The most important thing to remember when approaching a patient with religious hallucinations is that these experiences may have a massive impact and be all-consuming. Supporting patients to form trusting relationships with clinical staff and with their faith community may be paramount.

### Epistemic injustice

Epistemic injustice occurs when a person is given less credence than they deserve, often because of the prejudices of others. Philosophers applying Miranda Fricker's concept of epistemic injustice to psychiatry have focused on two key forms of injustice experienced by people with severe mental illness.^13–15^ Testimonial injustice is where a patient's testimony is not believed, found credible or sufficiently taken into account. Contributory injustice is where the epistemic resources needed to make sense of a marginalised individual's experience exist within the marginalised group but are not shared by the dominant group.^16^ The suffering arising from these forms of injustice can compound existing distress and be a barrier to the formation of trusting relationships. A patient with spiritually significant hallucinations can be misbelieved or misinterpreted in different ways by their friends, family or faith community and by the clinical staff trying to help them. This can be very upsetting, confusing and isolating. It thus threatens the patient's willingness or capacity to engage in psychiatric treatment.

A variety of strategies have been suggested to counter epistemic injustice. Drożdżowicz argued that healthcare professionals have a ‘*pro tanto* epistemic duty to attend to and/or solicit reports of patients’ first-person experiences in order to prevent epistemic losses’.^17^ Narrative can play a key role in this.^18,19^ Miller Tate suggests that it is incumbent on psychiatrists to develop their understanding of the wider social or religious group to which the patient belongs.^16^

### The trainee perspective (R.J.C.)

I was drawn to psychiatry as the specialty where I most got to spend time with patients, getting to know their stories and their lives. As you progress through training there is the very real pressure to learn and gain knowledge to pass exams and get to the next stage. It has been easy to get lost in the intellectual discussions about psychopathology and diagnosis and then to realise that I have not really understood what effect the patient's experience was having on them. I have lacked confidence in knowing how and when to ask patients about their religious/spiritual beliefs and there remain times when I forget to.

In the three scenarios above, the religious experiences are so central that to not discuss them would be difficult. I would want these patients to know that they can be open with me about all their experiences and be heard without judgement. As trainees we focus on building our relationships with patients. As we progress through training and into consultant posts, how we form these relationships becomes integral to how patients also form a relationship with the wider care team and mental health services.

## Practical management

### Spiritual assessment

Having someone show a keen and sincere interest in something so personally important should be affirming. However, we must remember that spiritually significant hallucinations are sensitive issues and a patient may need time to discuss these experiences. Experiences need to be listened to, validated and respected without passing judgement. This may be the first opportunity a person has had to freely share their experiences and it could be a huge relief for them to be taken seriously. As part of the history of the presenting complaint, take a history of the hallucinatory experiences, but also understand the impact these have had on the individual, their family and community, including asking about stigma and abuse. As part of the personal history, ask questions to understand how this person's religious and spiritual beliefs have developed over time – are there recent changes, is the impact of new experiences positive or negative? Are there patterns of positive or negative religious coping and who, if anyone, does the person turn to for advice and support for their spiritual needs.^20^ Explore the detailed content and interpretation of religious experiences with open questions and allow time and space for answers.

It could be argued that the primary purpose of history taking is therapeutic, and completion of the assessment to reach diagnosis and guide management are secondary goals. It is here that the key skills of a good psychiatrist are put into action – building a relationship of trust and being aware of epistemic injustices that might have occurred and where they may occur in this patient's journey. Without the foundation of trust and understanding between patient and clinician the whole process of treatment and recovery will be difficult or impossible.

### Formulation and diagnosis

Sims succinctly writes that ‘both mental illness and spiritual experiences are frequent in the population and so sometimes they coincide’.^21^ He and others have suggested reasons for deciding when spiritual experiences or behaviour are evidence of mental illness.^22^ However, approaching a patient purely to ascertain a differential diagnosis can perpetuate epistemic injustice. Instead, keep separate the important questions of diagnosis and spiritual meaning and consider both equally in every case. Diagnosis guides management but it should not invalidate all spiritual experiences. Alongside explanation and education around diagnosis, exploration with the patient of how these experiences have affected or continue to affect them is important.^23^

In this article, we have actively resisted giving strict guidelines about ‘how to tell when a spiritually significant hallucination is mental illness’, as this risks approaching a patient with a list of ‘tick boxes’, not listening to the narrative and ultimately inflicting epistemic injustice. It is recommended to ask early in the assessment what spiritual support the patient needs and to seek their consent to gain collateral history from family, friends and/or their spiritual leader. These enquiries can then establish whether there has been recent, rapid change in the content or strength of their beliefs, whether these are in keeping with the beliefs of their community and, if not, this allows for further discussion that could help to differentiate pathological thought processes from non-pathological spiritual beliefs.

### Addressing religious coping

There is a paucity of good research on outcomes following clinical interventions directed at improving religious coping, but affirming positive religious coping and talking with patients about their spiritual struggles (negative religious coping) are likely to be helpful where opportunity arises. A variety of psychotherapeutic strategies to address spiritual struggles are now available^24^ and, similarly, a range of treatment options are available to address religious hallucinations, particularly where these are distressing.^6^ The aim should be to reduce distress and affirm the constructive search for meaning within these experiences.

### Addressing epistemic injustice

It is the responsibility of the psychiatrist to understand the wider social and religious context of the patient's experiences.^16^ Chaplaincy services do not just support the patient but can also provide support and education for the clinician. It is important that we are all mindful of our own biases and limitations in knowledge, such that we prevent and/or avoid perpetuating the epistemic injustices often experienced by our patients.^15^ Swinton writes about the tendency for the experiences of people living with a diagnosis of schizophrenia to be seen simply as symptoms of their underlying brain disorder, thus having no real meaning for the individual or those around them.^25^ However, with true listening and stepping beyond epistemic injustice we can come together, seeing and hearing each other in more meaningful ways.

### Chaplaincy services

Chaplains have been described as the ‘true spiritual care specialists’.^26^ Although healthcare professionals have key roles in identifying and assessing spiritual needs in relation to the health of their patients, they may not be best placed to address them. ‘Pastoral care provision’ in in-patient psychiatric units has been viewed as beneficial, and potential harms can be mitigated by ensuring caregivers have training and work within clearly defined roles but are also integrated into the team.^27^ The clinician's role may therefore be to provide early assessment of a patient's spiritual needs, followed by referral to trained spiritual caregivers or the relevant spiritual healer for that patient.^28^ An alternative model of integration of spirituality into National Health Service (NHS) healthcare has also been proposed, with a multidisciplinary spirituality working group to promote good practice.^29^

Chaplaincy/spiritual care departments in the NHS vary and, although they aim to provide support for patients from any religious or spiritual background, resources are limited.^30^ Ongoing training and education, including in mental health, are part of the competencies outlined by the UK Board of Healthcare Chaplains, but detail on what knowledge is expected is not included.^31^

Opening dialogue (with consent) between the clinicians, chaplains and the patient's faith leaders/community helps everyone better understand the patient's religious/spiritual experiences and identify elements of their mental health problems that could be better understood by their faith community. Chaplains can provide a liaison between the patient, their faith community and mental health services. In scenario 1 the patient is asking for prayer, which can be seen as actively seeking spiritual support, and therefore it would be appropriate to offer chaplaincy support. In scenario 3 the patient is asking to see their imam and this would be a good opportunity to seek consent to speak with them, which could be done either with or without chaplaincy input.

At the service level, successful projects to ‘bridge the gap’ between healthcare and local faith communities^32,33^ should be replicated.

### Practical tips

Starting the conversation about spiritually significant hallucinations can be difficult and some practical tips and key questions are provided in Box 1.

Box 1 Practical tips and key questions in treating a patient with spiritually significant hallucinations

Assessment

- Build trust
- Focus on the patient's experience
- Do the hallucinations have a positive or negative emotional impact?
- Do the hallucinations have any impact on the patient's function and daily life?
- Does the voice have spiritual significance?
- Explore the patient's religious or spiritual history
- Obtain information about their faith community (if any) and ask for consent to speak with their faith leader
- Identify positive and negative religious coping strategies

Diagnosis and formulation

- Are there any other symptoms of mental illness?
- Don't rush
- Continue to build trust

Treatment considerations

- Collaborate with chaplains and/or faith leaders – with the patient's consent if speaking with those who know them or via chaplains to learn more about the faith beliefs
- Praying with patients is much contested and the boundaries are not as clear as may be first thought; if it is practised, then patient consent remains essential^34^
- Encourage positive religious coping
- Consider spiritually integrated therapies^5^

## Conclusions

Spiritually significant hallucinations are common but are not pathognomonic of mental illness. Careful history taking, with focus on understanding the patient's narrative, experiences and spiritual needs is important. We advocate offering chaplaincy support to all patients who experience such hallucinations in order that they benefit from the skills our chaplain colleagues have to offer in addressing their spiritual needs. However, we also emphasise the importance of the clinician's own self-awareness and awareness of epistemic injustice. Chaplains offer an important role in clinician support when seeing patients with complex spiritual and/or religious needs and in providing ongoing education and awareness, such that epistemic injustice within mental health services may be addressed. It may take time, sometimes years, for a patient to be able to fully explore what meaning, if any, their hallucinations have had for them.

## About the authors

**Rachel J. Cullinan** was, at the time of writing the article, a specialist trainee in old age psychiatry at Cumbria, Northumberland, Tyne and Wear NHS Trust, Newcastle upon Tyne, UK. She is now a consultant old age psychiatrist at Gateshead Health NHS Foundation Trust, Gateshead, UK. She is an associate researcher in the Translational and Clinical Research department at Newcastle University, Newcastle upon Tyne, UK. **Angela Woods** is Professor of Medical Humanities and Director of the Institute for Medical Humanities at Durham University, Durham, UK. **Joanna M.P. Barber** is an honorary researcher at Birmingham and Solihull Mental Health NHS Foundation Trust, Birmingham, UK. **Christopher C.H. Cook** is Emeritus Professor in the Institute for Medical Humanities at Durham University, Durham, and Chair of the Spirituality and Psychiatry Special Interest Group at the Royal College of Psychiatrists, London, UK.

## Data Availability

Data availability is not applicable to this article as no new data were created or analysed in this study.
